# How chimpanzees (*Pan troglodytes*) share the spoils with collaborators and bystanders

**DOI:** 10.1371/journal.pone.0222795

**Published:** 2019-09-23

**Authors:** Maria John, Shona Duguid, Michael Tomasello, Alicia P. Melis

**Affiliations:** 1 Dr Maria John Communications, Leipzig, Germany; 2 Department of Experimental Psychology, University College London, London, United Kingdom; 3 Department of Psychology and Neuroscience, Duke University, Durham, NC, United States of America; Universidade de São paulo, BRAZIL

## Abstract

Chimpanzees hunt cooperatively in the wild, but the factors influencing food sharing after the hunt are not well understood. In an experimental study, groups of three captive chimpanzees obtained a monopolizable food resource, either via two individuals cooperating (with the third as bystander) or via one individual acting alone alongside two bystanders. The individual that obtained the resource first retained most of the food but the other two individuals attempted to obtain food from the "captor" by begging. We found the main predictor of the overall amount of food obtained by bystanders was proximity to the food at the moment it was obtained by the captor. Whether or not an individual had cooperated to obtain the food had no effect. Interestingly, however, cooperators begged more from captors than did bystanders, suggesting that they were more motivated or had a greater expectation to obtain food. These results suggest that while chimpanzee captors in cooperative hunting may not reward cooperative participation directly, cooperators may influence sharing behavior through increased begging.

## Introduction

Sharing the spoils of cooperatively gained rewards is key to maintaining cooperation. Human societies often have very clear rules about how the products of collaborative hunting or foraging should be shared among the participants and the wider community [1]. Chimpanzees, one of our closest living relatives, are known to hunt in groups and share the meat amongst group members [2]. This behavior is of particular interest to understanding the evolution of human cooperation. In the following experiment we aim to investigate the factors influencing the distribution of cooperatively gained resources amongst chimpanzees.

Group hunting of medium-sized mammals is widespread across chimpanzee communities. There is variation between communities in many aspects of the hunt; including the preferred prey, the composition of hunting parties, and how a hunt unfolds (see [3] for a review) but, typically, on encountering potential prey (usually arboreal monkeys) one or more male chimpanzees will initiate the chase through the canopy and others may join. A successful hunt can result in capture of several prey, the largest portion of which the captor(s) retain. However, a significant proportion of the group get meat, including bystanders who did not take part in the hunt [3].

Boesch [4] found participants in the hunt in Taï National Parkgained access to more meat than bystanders and suggested that it was because they participated in the collaborative activity and captors or meat possessors rewarded their partners’ efforts. This implies that the possessor recognizes the merit of their collaborative partners, skills that are seen in human children from around the age of 3–4 (e.g. [5–7]. If sharing is determined by participation in the hunt it would suggest that chimpanzees have an enforcement mechanism to sustain cooperation in groups, and would explain why individuals will initiate a hunt even if they are unlikely to be the captors [8]. However, there is evidence from chimpanzees in Gombe National Park suggesting that sharing is primarily determined by begging [9]. Begging behaviors can take several forms, such as sitting next to the possessor and staring at the meat or reaching towards the carcass. The authors considered begging to be harassment when the beggar touched the carcass or possessor, and there is evidence suggesting that such begging reduces possessor’s feeding speed [9]. Although sharing is costly to the possessor it also produces an immediate gain by reducing harassment from others. There is also some evidence for sharing with preferred social or mating partners [10]. Some of these factors could result in the same distribution pattern as described by Boesch (4). For example: if hunters are closer to the captor at the time of capture than the average bystander they may gain more meat as a byproduct of proximity (opportunity). A more recent study (31) in the same chimpanzee population has also found evidence that participation in the hunt predicts access to meat afterwards. This study measured patterns of begging and harassment and found that both hunters and bystanders begged and harassed at similar levels (in this case the authors considered begging to be harassment as whenever begging resulted in a change in the possessor’s feeding behavior or posture, which differs from the definition described above which defined harassment as contact from the beggar to the possessor or meat). The study also discusses that the results are unlikely to be an artifact of hunters being more motivated or closer to the captor (31). However, proximity was not a variable analyzed in this study and which can be difficult to incorporate in an observational study. Thus, the aim of the present study is to investigate the proximate mechanisms underlying distribution patterns experimentally with captive chimpanzees.

Captive populations do not have the opportunity to hunt but they do share food, including desirable and rare items [11–14]. Previous experimental studies portray a complex relationship between sharing and cooperation. Melis and colleagues [15] found both that making food more monopolisable (more like captured prey) decreased cooperation success (Experiment 1) and that increased sharing between partners was associated with higher cooperative success (via tolerance; Experiment 2). In addition, two other studies looked specifically at the effects of collaboration on sharing behaviour. Hamann and colleagues [16] tested whether chimpanzees were more likely to share food they had obtained by working together (by operating an apparatus together) in comparison to food released by an experimenter. They did not find that cooperation increased the likelihood to share but the rate of sharing was very low overall and the mechanism that allowed sharing was quite different from what would occur in the wild where individuals are in close proximity to each other. Melis, Schneider & Tomasello [17] enabled more typical sharing behavior in another study. They manipulated whether chimpanzees acquired food through cooperation (the loose string task) or individual work. In addition, they manipulated the distance a potential recipient was at the point of ‘capture’, thereby testing one simple alternative explanation offered for the patterns of meat distribution described by Boesch (4). The clear finding was that cooperating had no effect on the amount of food a non-captor obtained. Instead, the non-captor’s proximity to the food at the point of retrieval predicted sharing. These studies would suggest that the division of the spoils is not influenced by the role an individual had in the acquisition process.

In the current study we seek to expand the study by Melis and colleagues [17] by investigating the sharing patterns between three individuals as opposed to the dyads tested in the original study. With three individuals the roles of interest—captor, helper and bystander- emerge naturally within the same interaction. This is more comparable to the hunting context in the wild in which there are several potential recipients present. The simultaneous presence of helpers and bystanders can also facilitate a comparison between the two roles, potentially making it easier for captors to choose with whom to share. If captors recognize and reward the role helpers play in acquiring the resource we expect helpers to gain access to more of the reward than bystanders. Therefore, we analyzed sharing and begging patterns after collaborative food acquisition with regard to the role individuals played (helper vs. bystander, excluding the “food captor”), and the proximity to the captor. We also ran a control (solo) condition, in which one chimpanzee alone obtained the food. The goal was to compare sharing levels with the helper individuals in the cooperation condition to those with the same individual in the solo condition when they were a bystander.

## Materials and methods

The research was approved by the management, board members and veterinarian at the Chimpanzee Sanctuary and Wildlife Conservation Trust (CSWCT). It was also approved by the Ugandan National Council for Science and Technology (EC635) and Uganda Wildlife Authority.

Thirteen unrelated chimpanzees (*M* = 14.77 years, *range* = 12 to 21 years; 8 male, 5 female) living at Ngamba Island Chimpanzee Sanctuary, Uganda (www.ngambaisland.com) participated in this experiment. The chimpanzees at the sanctuary (47 at the time of the current study) are orphans rescued from illegal pet and bushmeat trade and remain at sanctuary for their lifetime. They have access to 95 acres of secondary forest during the day. In the evening, they can choose to enter a roofed enclosure to sleep (approx. 140m^2^). The chimpanzees receive four feedings per day in addition to food available in the forest and ad libitum access to water. Testing takes place in the roofed enclosure in the morning and participation is voluntary. The chimpanzees were not deprived of food or water at any point during the study.

All subjects had previously participated in Melis, Schneider & Tomasello [17] and demonstrated their collaborative problem-solving abilities in several other previous experimental studies (e.g. [15, 17, 18]. They had all demonstrated that they understood the need to coordinate their behavior with that of their partner to succeed in the collaboration task used in this study. The chimpanzees were grouped into eight tolerant triads, based on the knowledge from long-term caretakers. Each individual was a member of up to two triads, see Table A in S1 File for triad composition.

**Fig 1:**
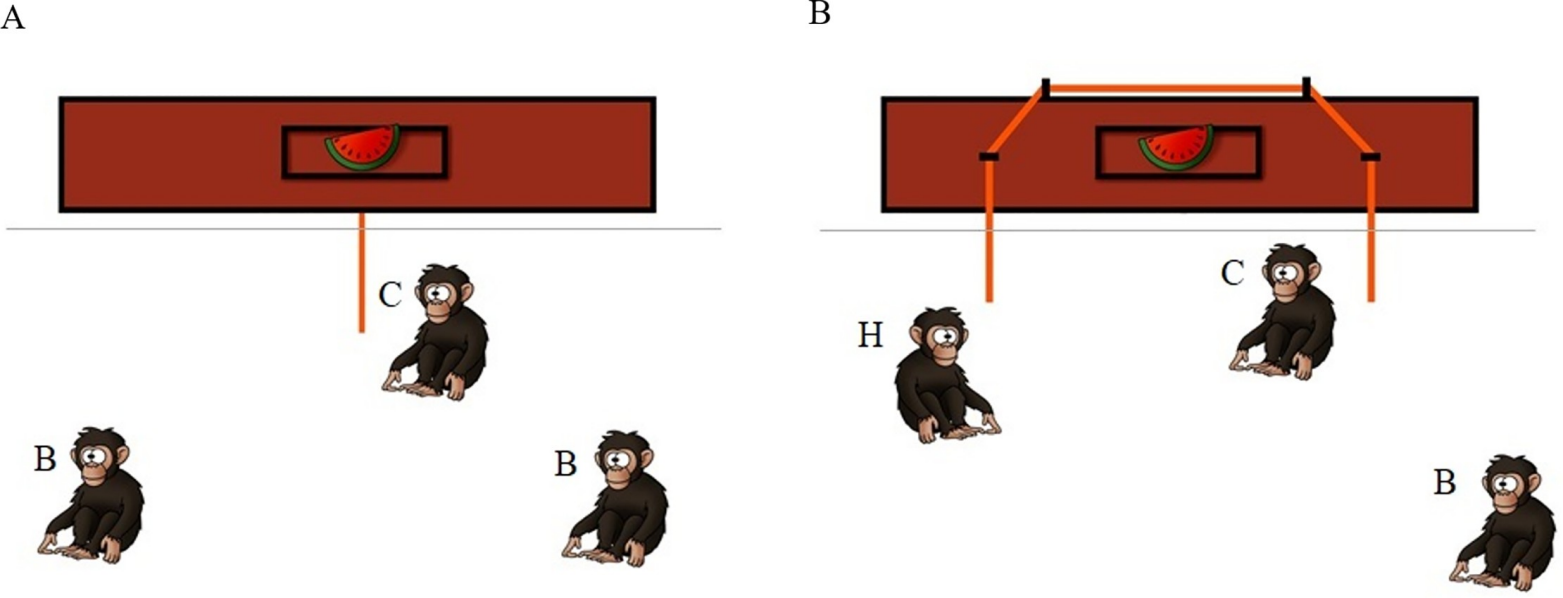
Experimental set-up for a) the solo condition and b) the cooperation condition. The individual who obtained the food was considered the captor (C); the other rope-pulling individual was considered the helper (H, Cooperation condition only); any individual not involved in food acquisition were considered bystander (B).

The apparatus was a flat 3.4m long platform with a food dish in the center, also used in previous collaborative problem-solving tasks (e.g. [15, 18]. The apparatus was placed 1 m outside the test enclosure, out of reach for the subjects (see Fig 1). The test enclosure was 5m x 3m x 4m.

Before the test phase, we performed dominance tests according to the methods in Melis and colleagues [17] to establish the dominance hierarchy within each triad. Each pair completed one session of six trials. The apparatus was set-up with a single rope and was baited with two banana slices. Two individuals entered the testing room together and the individual monopolizing the platform in the majority of trials determined dominance.

All triads participated in two experimental conditions: *solo* and *cooperation*. In both conditions, the platform was placed outside the testing enclosure and was baited with a monopolizable food item (half a watermelon or half a papaya). In the *solo* condition a rope (1.6 m) was attached to the center of the platform with the end placed inside the testing enclosure. All three individuals were allowed to enter the room simultaneously and one individual alone could pull the platform within reach. The individual who obtained the food was considered the “captor”; the other two were considered “bystanders” (Fig 1A).

In the *cooperation* condition a longer rope (5.8 m) was placed through the loops on top and across the length of the platform. Both ends of the rope extended from the platform through the metal bars into the test enclosure but were too far apart for one individual to pull both ends alone (approx. 3m apart). Again, all three individuals entered the room simultaneously. Two individuals needed to collaborate to pull the tray within reach. We considered the individual who pulled one end of the rope and obtained the food the “captor” (the individual first accessing the food was also always one of the two pulling the ropes); the other rope-pulling individual was considered the “helper”. The individual not involved in food acquisition was considered the “bystander” (see Fig 1B).

Each triad received six trials per condition but only one trial per day. The order of the conditions was randomized within triads across sessions, with the caveat that the same condition never occurred for more than two consecutive sessions. The type of food (either papaya or watermelon) alternated across sessions. Trials in which the triads did not succeed in pulling the tray were repeated a maximum of two times per session. If they did not succeed on the third attempt, the trial was considered unsuccessful and not repeated. Testing continued the following day with the next condition. If one individual was able to pull the tray in alone in the cooperation trial, the trial was cancelled and repeated after the triad was finished with all other test trials (8.33% of the cooperation trials were repeated for this reason).

### Coding

All trials were filmed and recorded with two non-stationary video cameras following the individuals handling of food. To quantify sharing we measured the time spent feeding (time spent chewing) in each trial. Analyses were conducted using the percentage of time feeding per trial. A trial lasted from the moment one individual touched the food for the first time until the last piece of food was consumed.

Additionally, we coded four different kinds of begging behavior following the categories used by Gilby (9):

- Sitting and staring: beggar is located within arms´ reach of the food possessor, bodily oriented towards him and staring at the fruit or possessor.
- Reaching: beggar reaches with hand/arm towards possessor/fruit or pouts lips towards the food, there is no physical contact.
- Holding: touching the food or possessor.
- Hand-Mouth: beggar places a hand directly over the possessor´s mouth.

As some categories were rare, analyses were conducted using the total percentage of time per trial each individual spent begging (the sum of all four begging categories).

We coded the distance from the food at the moment of ‘capture’ for each individual. For every trial each individual was assigned one of the following four levels: 0) touching the food–the captor was always assigned this level; 1) within arm’s reach of the food; 2) one to two arm’s lengths from the food; 3) more than two arm’s lengths away.

Inter-observer reliability was determined for a randomly chosen 25% of all trials, by a coder blind to the conditions and hypotheses being tested. Inter-observer reliability was good for the time individuals spent feeding (r_s_ = 0.92, *N* = 24, *P* < 0.001) and begging (r_s_ = 0.96 *N* = 24, *P* < 0.001).

### Analysis

To assess which factor influenced how much helpers and bystanders fed and begged, we conducted four general linear mixed models (GLMMs; [19]. The analyses were conducted in R v3.5.2 [20] using the function lmer of the R-package lme4 [21]. As an overall test of the predictor variables, and to minimize Type 1 errors, we compared the full model with a null model lacking the test predictors using a likelihood ratio test [22]. P values for the individual effects were based on likelihood ratio tests comparing the full with respective reduced models (R function drop1).

Captors were excluded from this analysis because they spent nearly the whole trial feeding and they never begged (since they were in possession of the food). The first two GLMMs investigated the (1) feeding behavior and (2) begging behavior of helpers and bystanders in the cooperation condition (only in the cooperation condition were there both helpers and bystanders present). A third GLMM (3) investigated the relationship between feeding behavior and begging in the cooperation condition by testing whether time spent begging predicted the time spent feeding in the cooperation condition. The final two GLMMs investigated the (4) feeding and (5) begging behavior of the helper individuals in the cooperation condition compared to the behavior of the same individual in the solo condition when they were a bystander. The response variable was either proportion of spent feeding or proportion of time spent begging. Rank, role (helper, bystanders) as well as proximity at the moment of food acquisition were predictor variables in all models. Additionally, we included trial number as a control variable; the identity of the individual and the identity of the triad as random effects; and a maximal random slopes structure [23]. See S1 File for further details of model construction.

## Results

All eight triads succeeded in pulling the tray in all solo condition trials, but some of the triads did not always succeed in the cooperation condition. In 5 out of 48 (10.42%) cooperation trials individuals failed to pull the rope simultaneously and the rope went out of reach.

Captors, i.e. the individual obtaining the food, consumed most of the food in both the solo and the cooperation conditions. The proportion of time they spent feeding was very similar in the solo (M(solo) = 83.05% of trial spent feeding) and cooperation conditions (M(cooperation) = 82.83% time). In 54% of all trials the captors were the most dominant individuals in the triad; Table 1 displays full details for captor rank across conditions. The captors were all males, except in the all-female triad. In the four triads where the bystander and helper roles were divided between one male and one female, males and females took both bystander and helper roles.

**Table 1:** 

	Rank			
Condition	1st	2nd	3rd	No Captor
Cooperation	0.44	0.27	0.19	0.10
Solo	0.56	0.25	0.19	NA

Proportion of trials as captor according to rank position within the triad. Trials in which there are no captor are failures to pull the rope together.

**Fig 2:**
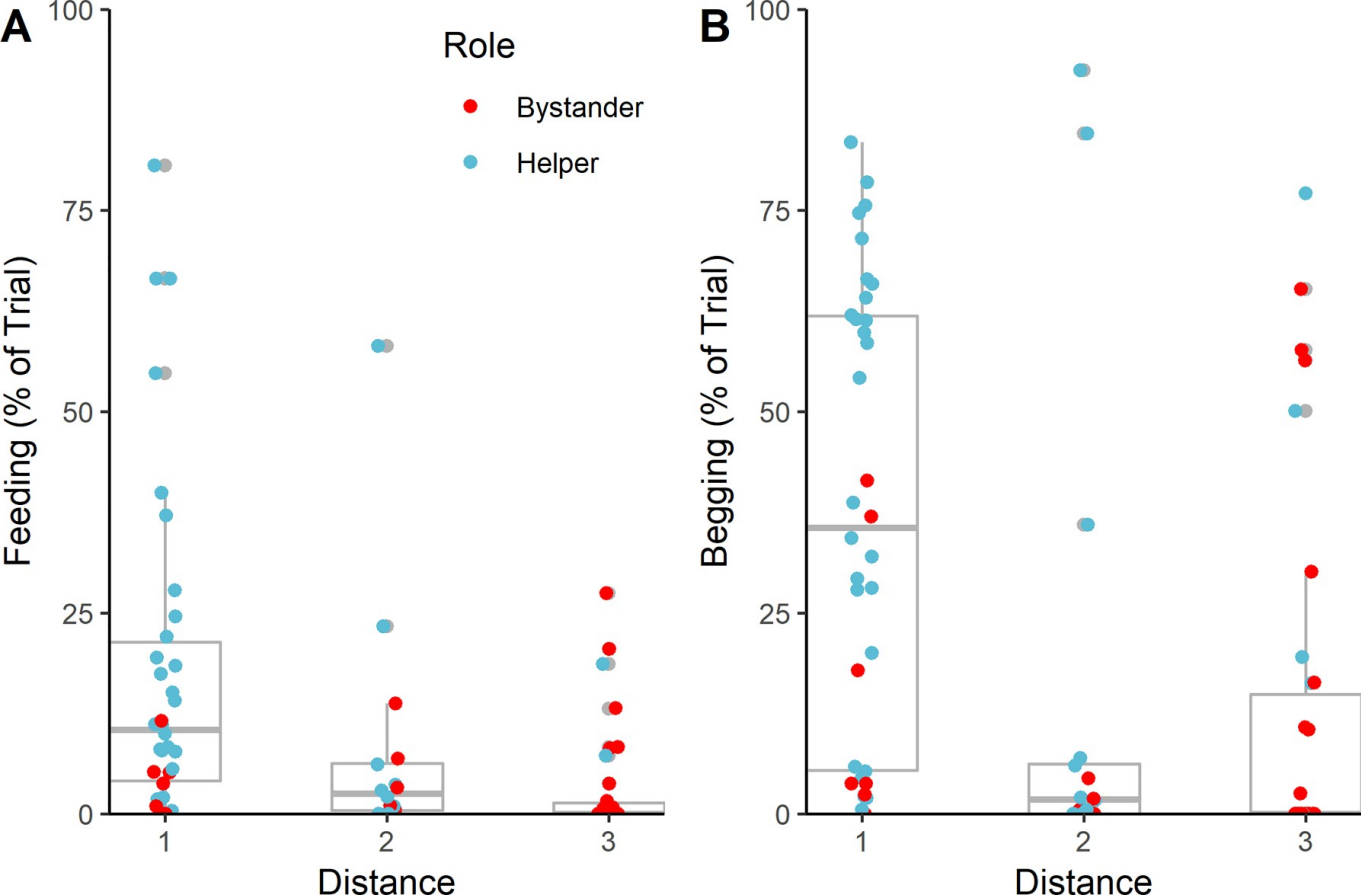
Percentage of trial spent feeding (A) or begging (B) and distance from food at the time of 'capture' (see Methods for details of distance coding). Data from helpers and bystanders only. Note this figure depicts the untransformed response variable. The lower and upper hinges correspond to the first and third quartiles (the 25th and 75th percentiles). The lower and upper whiskers extend from the hinge to no further than 1.5 times the interquartile range.

**Fig 3:**
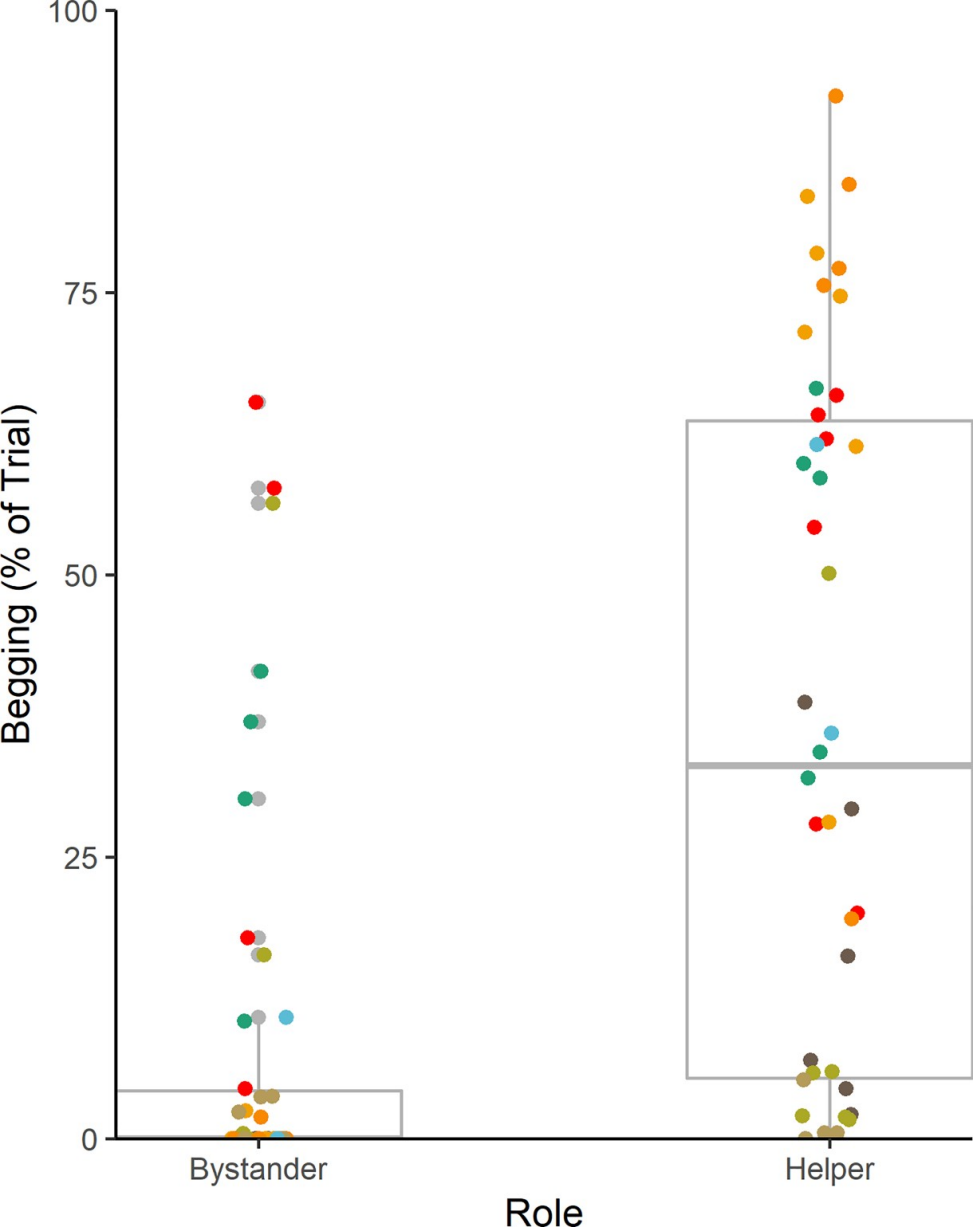
Percentage of trial spent begging by helpers and bystanders in the cooperation condition. Each color represents a triad. Note this figure depicts the untransformed response variable. The lower and upper hinges correspond to the first and third quartiles (the 25th and 75th percentiles). The lower and upper whiskers extend from the hinge to no further than 1.5 times the interquartile range.

Our main analysis concerned the behavior of helpers and bystanders in the cooperation condition. The main variable affecting the proportion of time feeding by non-captors (our proxy for amount of food obtained), was distance from the food at the moment of capture (see Fig 2A; estimate±SE = -0.46±0.14, χ = 5.40, df = 1, P = 0.02, N = 84 trials). Individuals that were closer in proximity to the captor and food spent more time feeding overall, supporting the findings of Melis et al. [17]. We did not find evidence that the role as helper or bystander, nor the individual’s rank, influenced the time spent feeding (see Tables B-F in S1 File) for full model results. Analysis of begging behavior indicated that helpers spent a higher proportion of time begging than bystanders did (see Fig 3; estimate±SE = 1.83±0.52, χ = 4.99, df = 1, P = 0.03). In addition, we found, that closer proximity also predicted an increase in time spent begging (see Fig 2B; estimate±SE = -0.33±0.14, χ = 3.85, df = 1, P = 0.049, N = 84 trials). In light of the finding that, in the cooperation condition, helpers spend more time begging than bystanders we ran further analysis to investigate whether begging leads to increased access to the food. To do this, we tested whether the addition of time spent begging in the cooperation condition improved the model of time spent feeding. We found that there was a trend towards increased time feeding with increased begging (estimate±SE = 0.42±0.20, χ2 = 3.54, df = 1, p = .06, N = 84).

We also compared how helpers behaved in the cooperation condition to how the same individuals behaved as bystanders in the solo condition. We found, as with the previous results, that increased proximity predicted increases in feeding and begging behavior (feeding: estimate±SE = -0.67±0.14, χ = 10.69, df = 1, P = 0.001, N = 115 trials; begging: estimate±SE = -0.29±0.14, χ = 6.64, df = 1, P = 0.01). Rank and role did not seem to influence either behavior although (but note that the GLMMs were not very stable with regard to these predictors; see S1 File materials for further details).

## Discussion

This study investigated sharing of cooperatively produced resources in semi-captive chimpanzees. Overall, we observed a typical pattern of sharing: the individual who first gains possession retains the vast majority of the resource but a proportion of it is shared, similar to other previously described situations with monopolisable resources in the wild [9, 10, 24–27] and in captivity [13, 14, 28]. Our main question was whether sharing favoured individuals helping to acquire the resource over bystanders. We found this was not the case: captors of the resource did not seem to differentiate sharing according to the role the recipient played in acquiring the resource.

Our results replicate previous findings by Melis and colleagues [17]; they also found that captors did not share more with whom they had collaborated to acquire the resource. We expected any preference for sharing with the collaborator to be more salient in the current study where the captor could choose directly between their collaborator (helper) and a bystander because they were tested in triads rather than dyads. However, in both the current experiment and Melis et al. [17], we have found that the main predictor of sharing was the physical proximity of the recipient to the captor at the acquisition moment and not their role in obtaining the resource. In other words, those that were at the resource first, were likely to gain more.

Although helpers were not rewarded for their role in acquiring the resource, they did beg significantly more than bystanders. This indicates that individuals expected more of the resource that they had worked to acquire and demand more from possessors. This increased begging could lead to a pattern in which collaborators do gain more of the resource, if increased begging leads to increased likelihood to share as has been observed with meat sharing [9]. Our results did suggest an overall trend in this direction and Melis et al. [17] also found a correlation between begging and increased sharing in three of the seven pairs they tested. This result emphasizes the importance of considering recipients’ expectations and behaviour in sharing patterns and further studies should aim to determine why helpers may be more motivated to get the food.

Naturally, there are differences between the cooperative task used in this experiment and group hunting that need to be acknowledged. The rope-pulling task in the current study is designed so that cooperation is necessary if the chimpanzees want to ‘capture’ the food and we have evidence from several experiments that these chimpanzees understood the role of the partner in the task (e.g. [15, 18, 29]. This could be a difference to hunting in wild communities, where a larger hunting party does increase the likelihood of hunting success, but cooperation is not strictly necessary as individuals do also hunt alone [3].This would predict captors in the current study should be more likely to share with their collaborators because of the essential role of their partner. On the other hand, although we did use large, monopolizable pieces of favored fruits, the chimpanzees in our study were ‘hunting’ for fruit rather than meat. There are examples of non-meat foods in the chimpanzee diet that are large and monopolizable or difficult to get, such as *Treculia africana* fruit or the proceeds of crop raiding, and these are more likely to be shared than other non-meat foods [24, 26] but there may be additional properties that change begging or sharing behavior for meat. For example, raw meat could simply allow for more time begging and sharing because it is more difficult and time consuming to eat than the fruits used for these captive studies. Additionally, although we tested triads of tolerant chimpanzees in the current study, in the wild captors have many beggars around them with whom they have different relationship qualities, which could also influence the sharing dynamics, motivating captors to share more, or more selectively, in order to avoid harassment. Finally, we recognize that we base our conclusions on 13 individuals from one social group. Given the variation in hunting behavior observed across chimpanzee communities in the wild (3), we hope future studies might determine whether the same mechanisms govern sharing and begging behavior in other groups.

The results from the current study, in combination with previous experimental findings [16, 17, 30], suggest that the instrumental act of collaborating in itself is not enough to prompt preferential sharing. Rather, sharing probably occurs through other proximate mechanisms. One important aspect that is repeatedly shown to be important in determining who gains from sharing is the simple factor of proximity–naturally, those who have opportunity are more likely to get access. This is also in line with the harassment hypothesis of sharing: meat possessors will share primarily to reduce harassment [9, 31]. A recent presented chimpanzees with the choice between delivering a food reward to themselves only, or delivering a reward to a partner as well as themselves. The authors found that chimpanzees were more likely to deliver food to a partner when that partner had made that choice possible in the first place by unlock a door. Especially when their partner paid a cost to unlock the door by forgoing an immediate food reward [32]. One important difference to the current study is that the food is not in physical possession of the chimpanzee at the time of making the decision. Furthermore, note that the study by Schmelz and colleagues does not create a context in which chimpanzees can/must choose between several individuals to share with (as is the case after group hunts in the wild and our current collaboration task). The study by Schmelz et al. shows that chimpanzees keep track of previous “favours” and reciprocate in dyadic interactions, but does not directly test rewarding mechanisms in a collective action scenario.

Our results complement and add to the most recent findings of meat-sharing in the wild [33]. Samuni et al. have found that hunt participants are more likely to get meat than bystanders. This would mean that, at a functional level, there is a mechanism in place that rewards participation in the hunts. However, this sharing pattern could come about through a different proximate mechanism from that in humans. One possibility could be that hunters beg more, either because they are more hungry or motivated, or more interestingly, because they expect to get part of the share after participation in the hunt. However, Samuni et al. found that hunters and bystanders begged at similar levels. Another possibility is that hunters tend to be closer allies and friends [10]. Finally, it is possible that hunters end up being closer to the meat possessor and therefore have more opportunities to get part of the spoils, so that it is proximity not hunting per se that drives the findings.

Samuni et al. also measured oxytocinergic system activity in hunt and hunting patrol participants and found increased levels after both hunting and patrols. This increased activity in the oxytocinergic system has also been associated with food sharing and social bonding in chimpanzees [27, 34]. The authors suggest that the increase in oxytocin during coordinated and cooperative activities such as hunting, hunting patrols, border patrols and food sharing [33, 35] create a positive feedback loop to prompt further prosocial behavior between those individuals. Further experimental studies could benefit from the addition of hormone measures to test whether cooperative and coordinated tasks in captive settings result in similar hormone level changes observed in the wild (and if this is not the case: why?), or if the lack of any changes could be one explanation for the difference in findings.

In summary, we do not find evidence in this experiment that chimpanzees preferentially share with those that helped them acquire the resource. This is in line with the results from previous experiments [16, 17] but in contrast to the positive effect of collaboration on sharing in humans from age 3 [7, 16, 36] and in a recent study with wild chimpanzees (31). Increased begging time did suggest that individuals helping to acquire the reward were more motivated or had a higher expectation of gaining some of the resource, a finding which deserves further investigation. We suggest that food-sharing in chimpanzees may be more strongly influenced by other factors such as opportunity, harassment avoidance and social bonds, and that findings that hunters are more likely to obtain meat than bystanders may be supported by different proximate mechanisms to those found in humans.

## Supporting information

S1 File Supplementary materials. Supplementary methods, analysis and results.
(DOCX)

S1 Data Data file. Supporting data files.
(XLSX)
