# Supplementary material for: How chimpanzees (Pan troglodytes) share the spoils with collaborators and bystanders
Source: PLoS One. 2019 Sep 23;14(9):e0222795. doi: 10.1371/journal.pone.0222795 (PMC6756536; doi:10.1371/journal.pone.0222795)
Supplement: S1 File — Supplementary methods, analysis and results. (DOCX) [file pone.0222795.s001.docx]

How Do Chimpanzees (*Pan Troglodytes*) Share the Spoils of Cooperation?

**Supplementary Material**

**Supplementary Methods**

Table A Triad composition and experimental experience

| Triad | Subject | Rank in Triad | Sex | Estimated Age (years) | Experimental history |
| --- | --- | --- | --- | --- | --- |
| 1 | Indi | 1 | Male | 13 | a, b, d, e, f |
|  | Baluku | 2 | Male | 14 | a, b, c, d, e, f |
|  | Namukisa | 3 | Female | 14 | a, b, c, d, e, f |
| 2 | Asega | 1 | Male | 14 | a, b, c, d, e, f |
|  | Kalema | 2 | Male | 16 | a, b, c, d, e, f |
|  | Okech | 3 | Male | 13 | a, b, c, d, e, f |
| 3 | Nkumwa | 1 | Female | 16 | b, c, d, e, f |
|  | Umutama | 2 | Male | 16 | b, c, d, e, f |
|  | Bili | 3 | Female | 14 | a, b, c, d, f |
| 4 | Umugenzi | 1 | Male | 15 | a, b, c, d, e, f |
|  | Yoyo | 2 | Female | 14 | b, c, d, e, f |
|  | Bwambale | 3 | Male | 12 | b, c, d, e, f |
| 5 | Yoyo | 1 | Female | 14 | b, c, d, e, f |
|  | Nkumwa | 2 | Female | 16 | b, c, d, e, f |
|  | Sally | 3 | Female | 21 | c, e, f |
| 6 | Kalema | 1 | Male | 16 | a, b, c, d, e, f |
|  | Umutama | 2 | Male | 16 | b, c, d, e, f |
|  | Namukisa | 3 | Female | 14 | a, b, c, d, e, f |
| 7 | Okech | 1 | Male | 13 | a, b, c, d, e, f |
|  | Indi | 2 | Male | 13 | a, b, d, e, f |
|  | Umugenzi | 3 | Male | 15 | a, b, c, d, e, f |
| 8 | Baluku | 1 | Male | 14 | a, b, c, d, e, f |
|  | Bili | 2 | Female | 14 | a, b, c, d, f |
|  | Bwambale | 3 | Male | 12 | b, c, d, e, f |

a: Melis, Hare, and Tomasello (2006a), b: (Melis, Hare, and Tomasello 2006b), c: Hare et al. (2007), d: Melis, Hare, and Tomasello (2008), e: Bullinger, Melis, and Tomasello (2011), f: Melis, Schneider, and Tomasello (2011)

Analysis

We used generalized linear mixed models (GLMM, (Baayen, Davidson, and Bates 2008)) with a gaussian error structure to analyse the food gained and begging behaviour. All models were fitted in R v3.5.2 (R Core Team, 2018) using the function *lmer* of the R-package *lme4* (Bates et al. 2015). All models followed the same structure: the test predictors were proximity and the interaction between role (helper or bystander) and relative rank and trial number as control predictor. Proximity, rank and trial number were all z-transformed. To keep type I error rate at the nominal level of 5% (Schielzeth and Forstmeier 2009, Barr et al. 2013) we included all possible random slopes components: subject and triad as random intercepts and all possible random slopes with proximity, trial number rank and role (including the random slopes of the interaction of role and rank with subject and triad). The categorical variable (role) were centred for the random slopes. We assessed model stability by comparing the estimates obtained from a model based on all data with those obtained from models with the levels of the random effects excluded one at a time. To rule out collinearity we determined Variance Inflation Factors (VIF, (Field 2005) for a standard linear model excluding the random effects. VIF values were below 2.0 for all models.

As an overall test of the effect of the fixed effects we compared the full model with a null model lacking the fixed effects but comprising the same random effects structure as the full model (Forstmeier and Schielzeth 2011). We used likelihood ratio tests (LRT; (Dobson 2002) to assess whether the inclusion of predictors improved the general fit of a model to the data. If this analysis suggested that the effects did improve the fit of the model, the individual contributions of the predictors were assed with LRT tests using the *drop1* function with the function test set to “Chisq” (Barr et al. 2013).

**Supplementary Results**

Model 1: Do helpers gain access to more food than bystanders?

The aim of the first model was to investigate whether helpers gain access to more food than bystanders. We did this by comparing the food acquired by helpers and bystanders in the cooperation condition. As a proxy for the amount of food, the response measure is the proportion of time individuals spend feeding per trial. The response variable was log-transformed prior to analysis (this applies to all subsequent models). The comparison of the full model with all test predictors and the null model was significant (χ2= 10.40, df=4, p=.034, N=84). The interaction term between rank and role did not significantly contribute to the overall model (χ2= 1.90, df=1, p=.17). Thus, we created a reduced model without the interaction term. Table B summarises this model. With increasing distance from the food at time of captor, the time spent feeding decreases.

Table B

|  | Estimate | SE | χ^2^ | df | p |
| --- | --- | --- | --- | --- | --- |
| Intercept | 1.19675 | 0.23829 |  |  |  |
| Rank | -0.06924 | 0.22317 | 0.0953 | 1 | 0.75756 |
| Role (Helper)* | 0.53082 | 0.36558 | 1.8444 | 1 | 0.17444 |
| Distance | -0.45913 | 0.14189 | 5.3921 | 1 | 0.02023 |
| Trial | 0.13264 | 0.10651 | 1.5020 | 1 | 0.22036 |

*Reference level: Bystander

Model 2: Do helpers beg more from captors than bystanders do?

The aim of this model was to investigate whether helpers beg more from captors

than bystanders do. We did this by comparing the proportion of the trial spent begging by helpers and bystanders in the cooperation condition. The comparison of the full model with all test predictors and the null model was significant (χ2= 12.40, df=4, p=.015, N=84). The interaction term between rank and role did not significantly contribute to the overall model (χ2= 1.44, df=1, p=.23). Thus, we created a reduced model without the interaction term. Table B summarises this model.

Table C

|  | Estimate | SE | χ^2^ | df | p |
| --- | --- | --- | --- | --- | --- |
| Intercept | 1.1391 | 0.4196 |  |  |  |
| Rank | 0.5885 | 0.1774 | 1.7108 | 1 | 0.19089 |
| Role (Helper)* | 1.8300 | 0.5193 | 4.9890 | 1 | 0.02551 |
| Distance | -0.3289 | 0.1434 | 3.8499 | 1 | 0.04975 |
| Trial | 0.1126 | 0.1111 | 0.8609 | 1 | 0.35348 |

*Reference level: Bystander

Model 3: Does begging predict access to more food?

The results of model 2 suggested that helpers were more likely to beg than bystanders in the cooperation condition. In light of these findings we ran some further analysis to investigate whether begging leads to increased access to the food. We did this by adding time spent begging as a predictor to the model of time spent eating in the cooperation condition (reduced model 1). The comparison of the full model with time spent begging as a predictor (z-transformed) and the null model including all predictors in model 1, removing only begging, indicated a tendency towards higher levels of feeding with increased begging (Estimate= 0.42, SE= 0.20, χ2= 3.54, df=1, p=.06, N=84).

Table D

|  | Estimate | SE | χ^2^ | df | p |
| --- | --- | --- | --- | --- | --- |
| Intercept | 1.21122 | 0.22033 |  |  |  |
| Rank | -0.09018 | 0.14799 | 0.3658 | 1 |  |
| Role (Helper)* | 0.53673 | 0.31291 | 1.7712 | 1 | 0.18323 |
| Distance | -0.35321 | 0.14494 | 4.6529 | 1 | 0.03100 |
| Trial | 0.10437 | 0.10444 | 0.9293 | 1 | 0.33504 |
| Begging Time | 0.41695 | 0.20470 | 3.5377 | 1 | 0.05999 |

*Reference level: Bystander


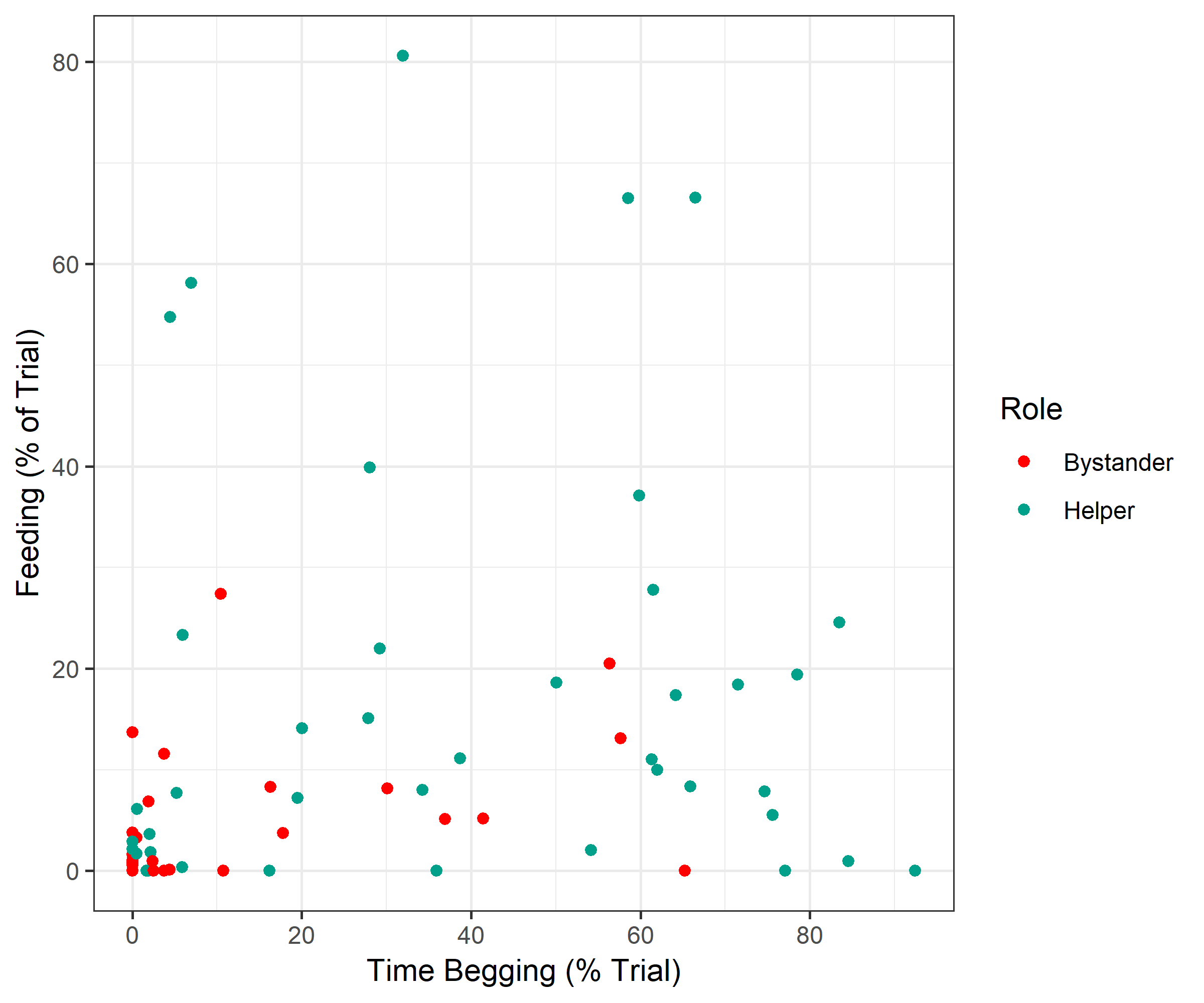


Figure A Scatterplot of the feeding against begging time in the cooperation condition. Note this is the raw, untransformed data and points represent one individual on one trial.

Model 4: Do individuals eat more when they are helpers than when they are bystanders?

The aim of this model was to investigate whether individuals gain access to more food when they act as helpers than when they act as bystanders. We did this by comparing the proportion of the trial spent eating by helpers in the cooperation condition to the same individuals as bystanders in the solo condition. The comparison of the full model with all test predictors and the null model was significant (χ2= 11.64, df=4, p=.020, N=115). The interaction term between rank and role did not significantly contribute to the overall model (χ2= 0.05, df=1, p=.83). Thus, we created a reduced model without the interaction term. Table E summarises this model. The model stability check indicated that the estimates for rank and role were subject to influential levels of the random effects (either the individual or the triad) thus we cannot draw strong conclusions from the estimates of rank and role in this model.

Table E

|  | Estimate | SE | χ^2^ | df | p |
| --- | --- | --- | --- | --- | --- |
| Intercept | 1.82526 | 0.16975 |  |  |  |
| Rank | -0.15361 | 0.24188 | 0.3885 | 1 | 0.533084 |
| Role (Helper)* | -0.15453 | 0.20998 | 0.5226 | 1 | 0.469715 |
| Distance | -0.65969 | 0.14397 | 10.6881 | 1 | 0.001078 |
| Trial | 0.07814 | 0.11646 | 0.4324 | 1 | 0.510824 |

*Reference level: Bystander


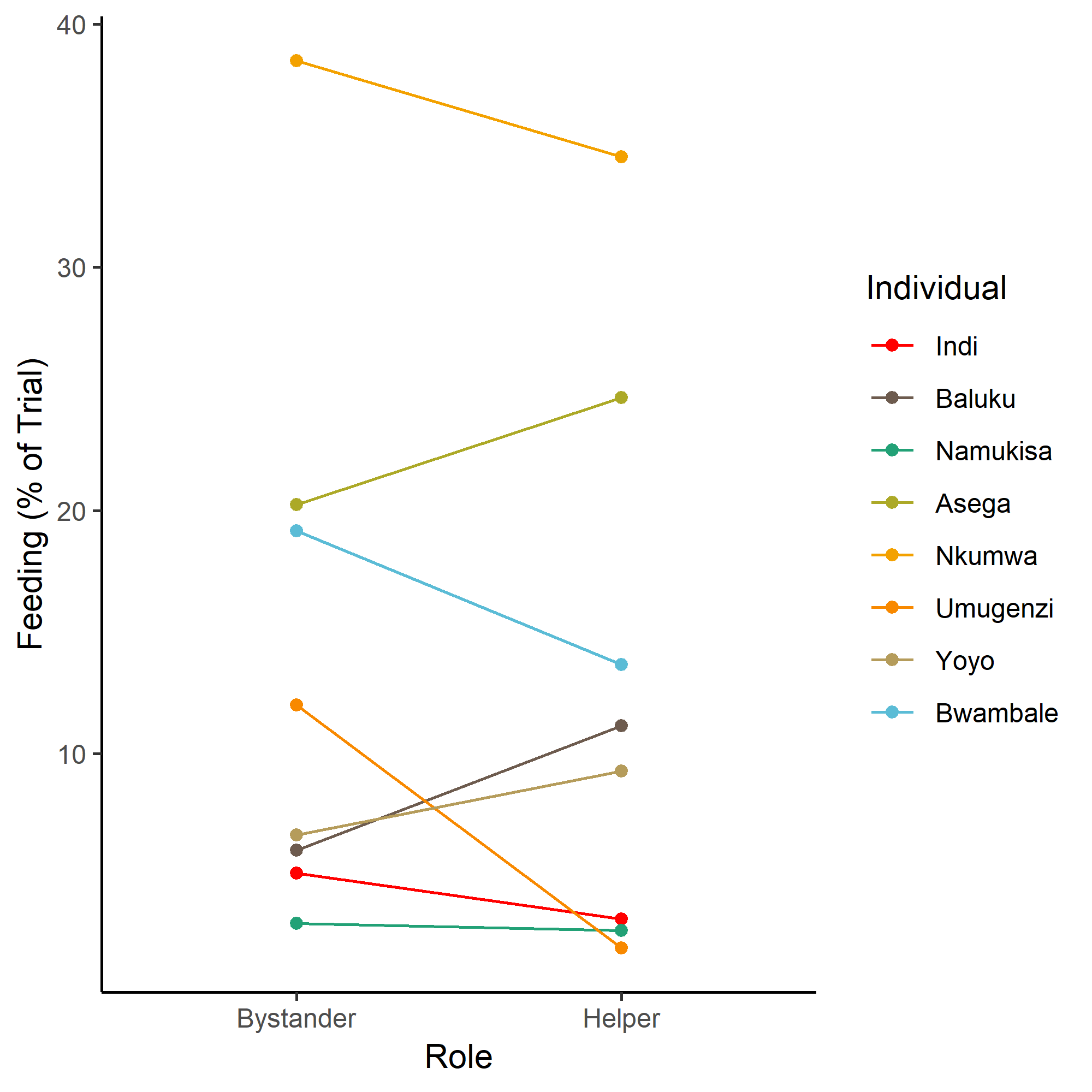


Figure B Mean percentage of trial spent feeding as helper in cooperation condition and non-helper in solo condition

Model 5: Do individuals beg more when they are helpers than when they are bystanders?

The aim of this model was to investigate whether individuals beg more when they act as helpers than when they act as bystanders. We did this by comparing the proportion of the trial spent begging by helpers in the cooperation condition to the same individuals as bystanders in the solo condition. The comparison of the full model with all test predictors and the null model was significant (χ2= 10.91, df=4, p=.028, N=115). The interaction term between rank and role did not significantly contribute to the overall model (χ2< 0.01, df=1, p=.98). Thus, we created a reduced model without the interaction term. Table F summarises this model. The model stability check indicated that the estimates for role and trial were subject to influential levels of the random effects (either the individual or the triad) thus we cannot draw strong conclusions from the estimates of role in this model.

Table F

|  | Estimate | SE | χ^2^ | df | p |
| --- | --- | --- | --- | --- | --- |
| Intercept | 2.3039 | 0.4001 |  |  |  |
| Rank | 0.4280 | 0.2110 | 3.2703 | 1 | 0.070543 |
| Role (Helper)* | 0.2864 | 0.2636 | 1.3307 | 1 | 0.248680 |
| Distance | -0.2880 | 0.1440 | 6.6362 | 1 | 0.009993 |
| Trial | 0.0110 | 0.1383 | 0.1870 | 1 | 0.665447 |

*Reference level: Bystander


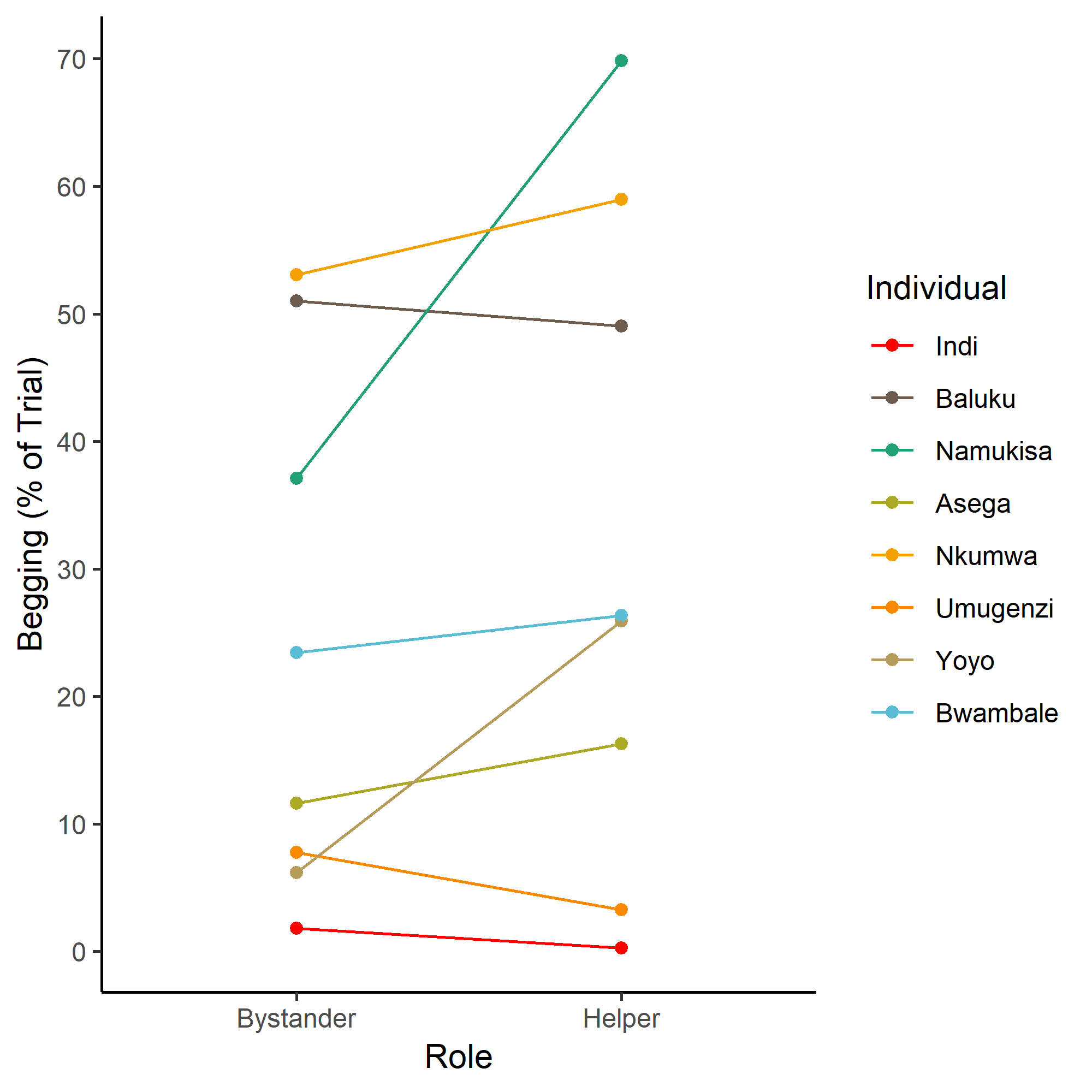


Figure C Mean percentage of trial spent begging as helper in cooperation condition and non-helper in solo condition

**References**

Baayen, R. H., D. J. Davidson, and D. M. Bates. 2008. "Mixed-effects modeling with crossed random effects for subjects and items." *Journal of Memory and Language* 59 (4):390-412. doi: DOI 10.1016/j.jml.2007.12.005.

Barr, Dale J, Roger Levy, Christoph Scheepers, and Harry J Tily. 2013. "Random effects structure for confirmatory hypothesis testing: Keep it maximal." *Journal of memory and language* 68 (3):255-278.

Bullinger, Anke F., Alicia P. Melis, and Michael Tomasello. 2011. "Chimpanzees, Pan troglodytes, prefer individual over collaborative strategies towards goals." *Animal Behaviour* 82 (5):1135-1141. doi: <http://dx.doi.org/10.1016/j.anbehav.2011.08.008>.

Dobson, Annette J. 2002. *An Introduction to Generalized Linear Models*. 2nd ed. Boca Raton, FL: Chapman & Hall/CRC.

Field, Andy. 2005. Discovering statistics using SPSS . Thousand Oaks, CA, US. Sage Publications, Inc.

Forstmeier, Wolfgang, and Holger Schielzeth. 2011. "Cryptic multiple hypotheses testing in linear models: overestimated effect sizes and the winner's curse." *Behavioral Ecology and Sociobiology* 65 (1):47-55.

Hare, Brian, Alicia P. Melis, Vanessa Woods, Sara Hastings, and Richard Wrangham. 2007. "Tolerance allows bonobos to outperform chimpanzees on a cooperative task." *Current Biology* 17 (7):619-623. doi: 10.1016/j.cub.2007.02.040.

Melis, Alicia P., Brian Hare, and Michael Tomasello. 2006a. "Chimpanzees recruit the best collaborators." *Science* 311 (5765):1297-1300. doi: 10.1126/science.1123007.

Melis, Alicia P., Brian Hare, and Michael Tomasello. 2006b. "Engineering cooperation in chimpanzees: tolerance constraints on cooperation." *Animal Behaviour* 72 (2):275-286.

Melis, Alicia P., Brian Hare, and Michael Tomasello. 2008. "Do chimpanzees reciprocate received favours?" *Animal Behaviour* 76 (3):951-962. doi: 10.1016/j.anbehav.2008.05.014.

Melis, Alicia P., Anna-Claire Schneider, and Michael Tomasello. 2011. "Chimpanzees, *Pan troglodytes*, Share Food in the Same way After Collaborative and Individual Food Acquisition." *Animal Behaviour* 82 (3):485-493. doi: 10.1016/j.anbehav.2011.05.024.

Schielzeth, Holger, and Wolfgang Forstmeier. 2009. "Conclusions Beyond Support: Overconfident Estimates in Mixed Models." *Behavioral Ecology* 20 (2):416-420. doi: 10.1093/beheco/arn145.
